# Advancing depression treatment: the role of sigma receptor ligands

**DOI:** 10.3389/fnins.2025.1691987

**Published:** 2025-11-25

**Authors:** Peng Zhang, Zeqiong Ning, Haixia Chen, Jia Chen, Zhihong Lu, Feifei Xu

**Affiliations:** 1 Department of Anesthesiology and Perioperative Medicine, Xijing Hospital, The Fourth Military Medical University, Key Laboratory of Anesthesiology (The Fourth Military Medical University), Ministry of Education, Shaanxi Provincial Clinical Research Center for Anesthesiology Medicine, Xi'an, China; 2 Department of Anesthesiology, Air Force Hospital of Western Theater Command, PLA, Chengdu, China; 3 Department of Pharmacy, Xijing Hospital, Fourth Military Medical University, Xi'an, China

**Keywords:** depression, sigma receptor, sigma receptor ligands, glutamatergic dysfunction, serotonergic system, Ca2+ responses, neuroinflammation, neuronal plasticity

## Abstract

Depression, one of the most prevalent neuropsychiatric disorders, continues to pose escalating global health challenges. Despite the discovery of numerous antidepressants, their clinical applications are limited, and there is still an urgent need to identify more effective antidepressant drugs and their molecular targets. In recent years, sigma receptors have garnered considerable interest among depression researchers because of their diverse biological functions and significant roles in the central nervous system. This review provides a comprehensive summary of the potential roles of sigma receptors and their ligands in the pathogenesis of depression and the course of antidepressant treatment, with the aim of offering insights into further research and potential therapeutic development.

## Graphical abstract

**Figure:**
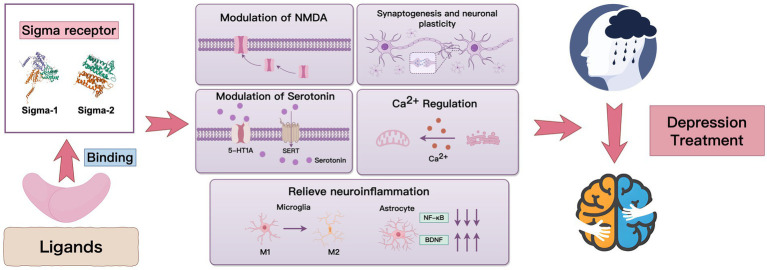

## 1 Introduction

Depression is a widespread mental disorder characterized by persistent sadness, lack of interest, low energy, concentration issues, and changes in appetite and sleep (Smith, 2014; Alexopoulos, 2005). It affects mental health, social life, and overall quality of life, with complex causes, including genetic, environmental, psychological, and biochemical factors (Monroe and Harkness, 2022; Rakel, 1999). Globally, over 300 million people are affected by this disease, making it a major contributor to the global disease burden (Lee et al., 2023). Its prevalence varies by country and is influenced by cultural, economic, and social factors (Malhi and Mann, 2018).

The etiology of depression is multifactorial and involves a complex interplay between genetic and environmental factors. Dysfunction of the brain’s monoamine neurotransmitter systems [e.g., 5-hydroxytryptamine (5-HT), norepinephrine (NE), and dopamine (DA)] (Shao and Zhu, 2020), immune-inflammatory responses (Yin et al., 2024; Han and Yu, 2014), imbalances in the hypothalamic–pituitary–adrenal (HPA) axis (Normann and Buttenschon, 2019), and dysregulation of the endorphin/*κ*-opioid receptor system (Wang et al., 2023) are considered critical contributors to depression. Despite advancements in the understanding of the pathological mechanisms of depression and the development and application of numerous new treatments, pharmacotherapy remains the primary modality for managing this condition (Sinyor, 2019). As the primary pharmacological agents of choice in clinical practice, selective serotonin reuptake inhibitors (SSRIs) represent a prevalent class of antidepressant medications that alleviate depressive symptoms by augmenting serotonin levels in the brain (Fu et al., 2024). Nevertheless, its clinical applicability is constrained by its adverse effects, but is not limited to sexual dysfunction, hyponatremia, cardiovascular events, elevated risk of fractures, and dermatological reactions (Zahiroddin et al., 2015; Varela Pinon and Adan-Manes, 2017; Kim et al., 2019; Khanassov et al., 2018; Masuka et al., 2022). Other frequently used pharmacological agents, including tricyclic antidepressants (TCAs), selective serotonin reuptake inhibitors (SSRIs), and monoamine oxidase inhibitors (MAOIs), have limited efficacy and delayed therapeutic action onset (Wang et al., 2022).

Recently, emerging studies have revealed that sigma receptors, a class of receptors extensively distributed throughout the central nervous system, are involved in the pathogenesis of depression by interacting with monoaminergic and glutamatergic pathways (Kulkarni and Dhir, 2009; Ren et al., 2025; Fujii et al., 2024; Izumi et al., 2024). They are promising therapeutic targets for the treatment of depression, attributable to their diverse structures and high binding affinities (Ren et al., 2025; Hashimoto, 2015; Garces-Ramirez et al., 2011). Drawing on preclinical studies and clinical trials, we provide a comprehensive review of the underlying mechanisms by which sigma receptors are involved in the pathogenesis of depression and emphasize the potential of sigma receptor ligands as therapeutic targets for depression.

## 2 Sigma receptors

Sigma receptors were originally proposed as a subtype of opioid receptors (Martin et al., 1976); however, further investigations have shown that they are specialized proteins that remain highly conserved among various species, cell types, and organelles (Hanner et al., 1996; Kekuda et al., 1996; Seth et al., 1997; Seth et al., 1998; Mei and Pasternak, 2001). In particular, the brain exhibits notable concentrations of sigma receptors within the hippocampus, frontal cortex, hypothalamus, olfactory bulb, and depression-associated regions such as the limbic and endocrine (Drevets et al., 2008; aan het Rot et al., 2009; Belmaker and Agam, 2008; Alonso et al., 2000). Sigma receptors are predominantly categorized as sigma-1 and sigma-2 receptors, which exhibit distinct structural and functional characteristics. The sigma-1 receptor (σ1R) is a pharmacologically regulated integral membrane protein predominantly located in the endoplasmic reticulum-mitochondrial-associated membrane (MAM). It is a highly conserved 223 amino acid protein with two transmembrane domains, including amino acid sequences 11–29 (transmembrane domain I) and 92–112 (transmembrane domain II) (Hanner et al., 1996; Kekuda et al., 1996; Su et al., 2016). In the central nervous system, the sigma-1 receptor has been reported to regulate various depression-associated processes, including synaptic plasticity, endoplasmic reticulum stress, neuroinflammation, and calcium homeostasis (Yang et al., 2019; Su et al., 2010; Hayashi and Su, 2007; Mori et al., 2013; Jia et al., 2018), by partially interacting with G protein-coupled receptors and ion channels. The sigma-2 receptor (σ2R) was recently characterized as a transmembrane protein 97 (TMEM97) (Alon et al., 2017). Extensive research has indicated that the sigma-2 receptor is overexpressed in various tumor cell lines, and its ligands demonstrate substantial efficacy in inhibiting cancer cell proliferation and survival (Oyer et al., 2019). Notably, a recent study reported that the activation of the sigma-2 receptor exhibits antidepressant activity comparable to that of citalopram and imipramine in a chronic mild stress model in mice (Sanchez and Papp, 2000), pointing to a novel direction investigating sigma-2 receptors in nervous system diseases.

## 3 Sigma receptor ligands

In 1994, Glennon et al. investigated the structure-affinity relationships of phenylalkylamine derivatives with respect to sigma-1 receptor binding. They identified super-potent sigma-1 ligands characterized by a pharmacophoric binding mode that includes a central basic amine nitrogen atom flanked by two hydrophobic features (Glennon et al., 1994). Since then, various unrelated and structurally different ligands have been found to exhibit high affinities for the sigma-1 receptor. These substances include anti-Parkinsonian amantadine (Kornhuber et al., 1993), analgesic pentazocine (Zhao et al., 2014), and antidepressant agents such as fluoxetine (Safrany and Brimson, 2016) (Table 1). To address this diversity, it has been proposed that sigma-1 receptors possess adaptable structures that enable binding to a wide range of structurally diverse compounds. However, the absence of a specific methodology has hindered our understanding of the mechanisms underlying these diverse ligand-receptor interactions. To address this issue, a new technique was recently developed. A novel set of BRET (Bioluminescence Resonance Energy Transfer) assays was developed to analyze how ligands induce multimerization of the sigma-1 receptor and its interaction with the immunoglobulin heavy chain-binding protein (BiP). The interaction between the heteromeric sigma-1 receptor and BiP, as examined using BRET assays, demonstrated that (+)-pentazocine and haloperidol caused opposite signal patterns (Yano et al., 2018).

**Table 1:** Representative compounds with affinity for sigma receptors.

Compounds	*K_i_* (nM)			1/2 ratio	Chemical class
	Sigma	Sigma-1	Sigma-2		
Desipramin (Narita et al., 1996)		343	2,107	0.163	TCAs
Imipramine (Narita et al., 1996)		1,987	11,430	0.174	TCAs
Opipramol (Rao et al., 1990)	50				TCAs
Sertraline (Narita et al., 1996)		57	5,297	0.095	SSRI
Citalopram (Narita et al., 1996)		292	5,410	0.054	SSRI
Paroxetine (Narita et al., 1996)		1,893	22,870	0.083	SSRI
Fluvoxamine (Martin et al., 2020)		36	8,439	0.004	SSRIs
Fluoxetine (Narita et al., 1996)		120	5,480	0.022	SSRIs
Clorgyline (Itzhak et al., 1991)		2.9	505	0.006	MAOIs
(+)Deprenyl (Itzhak et al., 1991)		82	1880	0.044	MAOIs
Harmaline (Itzhak et al., 1991)		310	2,100	0.148	MAOIs
Ro11-1163 (Itzhak et al., 1991)		860	>50,000	<0.0172	MAOIs
Progesterone (McCann et al., 1994)		246	15,700	0.016	Steroids
Deoxycorticorsterone (Su et al., 1988)	938				Steroids
Dehydroepiandrosterone (Takebayashi et al., 2004)	3,700				Steroids
Donepezil (Kato et al., 1999)	14.6				Acetylcholinesterase inhibitor
4-IBP (Megalizzi et al., 2007)		1.7	25.2	0.067	Derivatives of benzamide
PD144418 (Akunne et al., 1997)		0.08	1,377	0.00005	Oxalate
DTG (Lever et al., 2006)		35.45	39.87	0.889	Guanidines
SA4503 (Lever et al., 2006)		4.6	63.1	0.073	Piperazines
Igmesine (Earley et al., 1991)		39	390	0.100	Benzenamine hydrochloride
YL-0919 (Ren et al., 2023)		175.1	38,200	0.005	Pyridinone hydrochloride
BD 1047 (Matsumoto et al., 1995)		0.93	47	0.019	Derivatives of dichlorophenyl
BD 1063 (Matsumoto et al., 1995)		9.15	449	0.020	Derivatives of methylpiperazine
PRE-084 (Su et al., 1991)		2.2	13,091	0.0002	Phenylalkyl esters
PB190 (Skuza et al., 2014)		0.42	36.3	0.012	Derivatives of naphthalene
PB212 (Skuza et al., 2014)		0.03	17.9	0.002	Derivatives of naphthalene
Haloperidol (Lever et al., 2006)		0.90	7.93	0.113	Butyrophenones
NE-100 (Nakazato et al., 1999)		1.5	84.6	0.018	Propylamine hydrochloride
EST73502 (Garcia et al., 2020)		118			4-aryl analogs
EST64454 (Diaz et al., 2020)		22			Derivatives of pyrazolone
SI 1/28 (Wilson et al., 2022b)		6.1	583	0.010	Derivatives of methylpiperazine
LMH-2 (Deciga-Campos et al., 2020)		17	6		Butyrophenones
Dextromethorphan (Musacchio et al., 1989)		43	1,100	0.039	Methylmethylphosphates
(+)-Pentazocine (PTZ) (Lever et al., 2006)		1.62	728.4	0.002	Opioids
TS-157 (Shi et al., 2021)		1.8	304	0.006	Alkoxyisoxazole
Fenfluramine (Martin et al., 2020)	266				Amphetamines
SCH 23390 (Zhang G. F. et al., 2024)		3.16			Benzazepines
WLB-87848 (Christmann et al., 2024)		9	>1,000	<0.009	Thienopyrimidines
AF710B (Fisher et al., 2016)		250	>10,000	<0.025	Derivatives of diazaspiro
LS-1-137 (Malik et al., 2015)		3.2	256	0.013	Benzylpiperidine
RC-752 (Rossino et al., 2023)		6.2	360	0.017	Arylbutylamines
PB-28 (Berardi et al., 2009)		0.68	0.38	0.179	Cyclohexylpiperazines
Lu 28–179 (Perregaard et al., 1995)		17	0.12	141.667	Aminomethylindoles
CM572 (Nicholson et al., 2015)		>10,000	14.6	>684.93	Isosulfocyanides
CM398 (Wilson et al., 2022a)		>430	0.43	>1,000	Benzimidazolone
BS148 (Franchini et al., 2017)		62.8	2.5	25.12	Disulfiramocyclopentane
UKH-1114 (Sahn et al., 2017)		1,379	64	21.547	Methanobenzazocine
DKR-1005 (Sahn et al., 2017)		2,224	157	36.459	Methanobenzazocine
DKR-1051 (Sahn et al., 2017)		556	61	9.115	Methanobenzazocines
SAS-0132 (Sahn et al., 2017)		396	90	4.400	Norbenzomorphan
CT1812 (Rishton et al., 2021)			8.5		Isoindoline
2-Aminopyridine derivatives (Abate et al., 2012)		68.0	16.1	4.224	2-Aminopyridine
SM21 (Matsumoto et al., 2007)		1,050	145	7.241	Agmatine
AC927 (Matsumoto et al., 2007)			0.34		Phenethylpiperidines
UMB24 (Matsumoto et al., 2007)		322	170	1.894	Phenethylpiperazines
AD164 (Dichiara et al., 2022)		94	1,125	0.083	–
Memantine (Peeters et al., 2004)		2,600			Adamantanes
Amantadine (Peeters et al., 2004)		7,440			Adamantanes
Dimemorfan (Chou et al., 1999)		151	4,421	0.034	Methyl morpholans

*Only opipramol has been approved for the clinical treatment of depression (Le et al., 2024); the other ligands are currently in experimental stages.

## 4 Sigma receptors in depression: key mechanisms

### 4.1 Sigma receptors modulate glutamatergic dysfunction in depression

#### 4.1.1 σ1R-NMDA interactions

**Figure 1:**
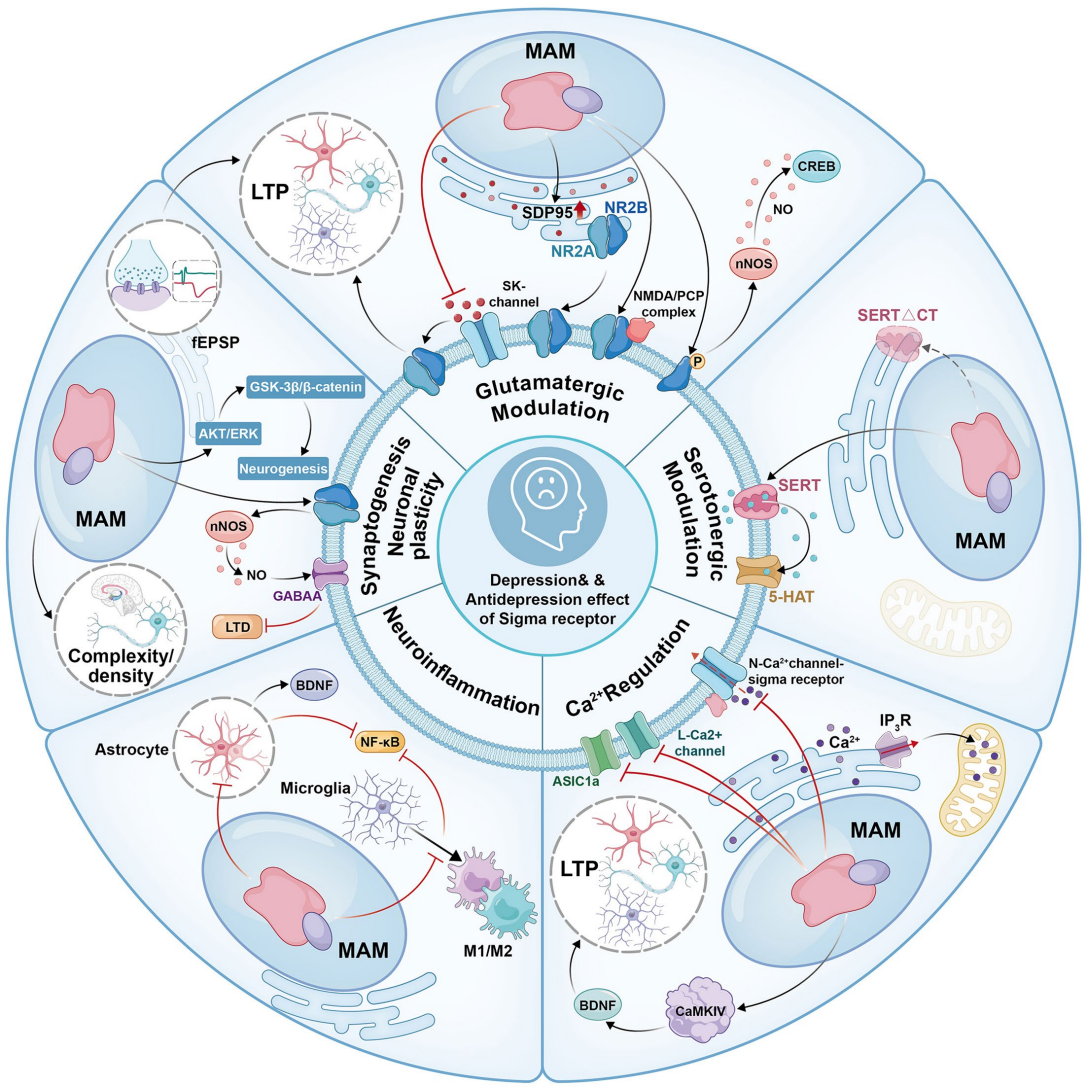
Intracellular signaling pathways in depression and the antidepressant effects mediated by sigma receptors.

Dysfunction in glutamatergic neurotransmission has been widely implicated in the pathogenesis of depression (Fan et al., 2023; De Berardis et al., 2020; Ferrero and Cereseto, 2004; Chu et al., 2021; Panczyszyn-Trzewik et al., 2023; Bagot et al., 2015; Shen et al., 2019). Compounds that modulate glutamatergic neurotransmission, including N-methyl-D-aspartic acid (NMDA) receptor antagonists, present a promising avenue for more effective and rapid treatment of depression compared to conventional monoaminergic antidepressants (Kadriu et al., 2019; Iosifescu et al., 2022; Tabuteau et al., 2022). Recent studies have revealed that sigma receptors can regulate neural activity mediated by NMDA receptors upon modulation by ligands (Pabba et al., 2014; Rodriguez-Munoz et al., 2018; Martina et al., 2007; Wang et al., 2007). For example, activation of the sigma-1 receptor can increase the expression of NMDA receptor subunit (NR2A and NR2B), promote NMDA receptor translocation to the cell surface, restore NR2B subunit phosphorylation in NMDA receptors by boosting the neuronal nitric oxide synthase (nNOS)—nitric oxide (NO)—cAMP-response element binding protein (CREB) signaling pathway and upregulate the phencyclidine (PCP)/NMDA receptor complex (Pabba et al., 2014; Zhang S. et al., 2017; Itzhak, 1994). In electrophysiology, activating the sigma-1 receptor can enhance NMDAR responses and long-term potentiation (LTP) by inhibiting Ca^2+^-activated K^+^ channels (SK channels), highlighting their role in synaptic transmission (Martina et al., 2007). Consistently, sigma-1 receptor knockout in mice impairs NMDA receptor-dependent LTP and independent long-term depression (LTD) in the basolateral amygdala, potentially leading to depression-like behavior (Zhang B. et al., 2017). These findings suggest that sigma receptor modulation of NMDA responses may contribute to the antidepressant-like effects (Figure 1).

#### 4.1.2 Ketamine synergy

Ketamine, a non-competitive NMDA receptor antagonist, is well known for its rapid antidepressant effects in patients with treatment-resistant depression. Some preliminary evidence suggests that activation of the sigma-1 receptor, in conjunction with ketamine, could produce significant outcomes that manifest within hours post-administration, whereas inhibiting the sigma-1 receptor significantly impairs ketamine’s ability to aid recovery in medial prefrontal cortex (mPFC) pyramidal neurons (Salvadore and Singh, 2013), indicating that the sigma-1 receptor may be involved in the lasting antidepressant effect of ketamine, and using ketamine with sigma-1 receptor agonists might improve the treatment for depression (Ma et al., 2024).

### 4.2 Sigma receptors interplay serotonergic system in depression

Evidence indicates that chronic stress impairs serotonin neurotransmission and reduces neurogenesis in the hippocampus, both of which are associated with the development of depressive symptoms. A diminished capacity for serotonin release in individuals undergoing major depressive episodes has been observed using positron emission tomography (PET) (Erritzoe et al., 2023). Currently, serotonergic neurotransmission is considered to be fundamental to the therapeutic efficacy of SSRI, a widely used treatment for major depressive disorder (MDD) treatment (Fried et al., 2015; Hale et al., 2013; Vahid-Ansari et al., 2019; Roberts et al., 2020), which mitigates depressive symptoms by reinstating serotonin levels and enhancing neurogenesis (Mahar et al., 2014). Interestingly, the effects of serotonergic modulators on depression are influenced by sigma receptors, although the effects of sigma receptors are pleiotropic. For example, in dizocilpine-induced cognitive impairment models, inhibition of sigma-1 receptors is reported to reduce the neuroprotective effect of a neuronal serotonin releaser, fenfluramine (N-ethyl-α-methyl-3-(trifluoromethyl) phenethylamine) (Martin et al., 2022). Consistently, activation of both sigma-1 and 5-HT1A receptors in a forced swimming test produced antidepressant-like effects, which were not observed when each receptor was activated individually. Inhibiting these receptors partially reduced the combined effect, indicating a synergistic antidepressant effect from simultaneous activation (Skuza and Rogoz, 2007). The sigma-1 receptor may enhance the membrane transport and uptake functions of the serotonin transporter (SERT) and its C-terminal deletion mutant (SERTΔCT) (Asano et al., 2019). These results indicate that sigma receptors function as neuroprotectants. Nevertheless, in DBA/2 mice, the sigma-1 receptor inhibition counteracted the reduction in fluvoxamine-induced immobility and functioned as an SSRI, consistent with the activation of the sigma-1 receptor resulting in a dose-dependent reduction in immobility (Sugimoto et al., 2012). These results indicate that sigma receptors may be neurotoxic. Additionally, modulating sigma receptors can impact serotonin-related gene transcription, influence stress responses, and possibly enhance the effectiveness of antidepressants (Skuza et al., 2011). These findings underscore the key role of sigma receptors in serotonin regulation, suggesting new pathways for antidepressant development via 5HT1A and sigma receptor activation.

### 4.3 Sigma receptors regulates Ca^2+^ responses in depression

Multiple Ca^2+^-dependent processes have been shown to contribute to the mechanisms underlying the antidepressant effects in animal behavioral models. The antidepressant effects of igmesine rely on both intracellular and extracellular calcium, consistent with the observation that the calcium chelator EGTA inhibited the antidepressant effects of igmesine and demethylimidazole. A recent study also suggested that mice lacking Orai1, a calcium channel, exhibit decreased inflammation-related Ca^2+^ signaling in astrocytes, inhibited neurotransmission in the hippocampus, and improvements in depression-like behaviors, including anhedonia and helplessness (Novakovic et al., 2023). Additionally, Kun Li et al. observed a significant increase in the levels of βCaMKII (calmodulin-dependent protein kinases that are essential for Ca^2+^ signaling) in the lateral habenula (LHb) in depression models. Lowering βCaMKII levels or inhibiting its activity can alleviate depression symptoms, making βCaMKII vital for LHb function and a significant factor in depression (Li et al., 2013).

Previous studies have shown that Ca^2+^-responses in depression are regulated by sigma receptors. Hayashi and Su (2007) showed that the sigma-1 receptor acts as a Ca^2+^-sensitive chaperone, aiding Ca^2+^ signaling between the endoplasmic reticulum (ER) and mitochondria during ER calcium depletion or ligand stimulation, thereby affecting cell survival. Moreover, sigma receptors also modulate various types of calcium channels, including (1) N-type calcium channel, the sigma-1 receptor is co-expressed with the N-type calcium channel in cholinergic interneurons of the rat striatum, and activation of the sigma-1 receptor significantly inhibited the N-type calcium current, whereas a sigma-1 receptor antagonist completely reversed this effect (Zhang K. et al., 2017); (2) L-type calcium currents (ICA-L), sigma-1 receptor activation inhibits ICA-L, which is blocked by sigma-1 receptor inhibition (Guo Y. et al., 2021; Chen et al., 2020); (3) voltage-gated ion channels (VGICs) within the Ca^2+^ superfamily, sigma-1 can directly modulate VGICs within the Ca^2+^ superfamily (Aishwarya et al., 2021), thereby regulating neuronal activity, including synaptic transmission and intrinsic excitability; (4) non-voltage-gated Ca^2+^-permeable channels that are regulated by sigma receptors through direct protein–protein interactions, including interactions with inositol 1,4,5-trisphosphate (IP3) receptors located in the endoplasmic reticulum, as well as with acid-sensing ion channel 1a (ASIC1a) in the plasma membrane (Mari et al., 2015). Prolonged activation of this sigma-1 receptor improves depression-like behaviors in CAMKIV null mice, increases hippocampal brain-derived neurotrophic factor (BDNF) mRNA, and enhances LTP, suggesting that sigma-1 antidepressant effects may involve the regulation of intracellular Ca^2+^ levels (Moriguchi et al., 2015). Overall, the direct modulation of calcium ion channels by sigma receptors represents a significant mechanism by which these receptors act as therapeutic targets for depression.

### 4.4 Sigma receptors regulate neuroinflammation in depression

Persistent neuroinflammation within the central nervous system (CNS), a multifaceted process involving various cellular entities, including microglia and astrocytes, plays a substantial role in the development of MDD (Woelfer et al., 2019; Liu C. H. et al., 2019; Zhang Y. et al., 2024; Kwon and Koh, 2020). Microglia are immune cells in the CNS that control neuroinflammation (Salter and Beggs, 2014). Previous studies have shown that activating sigma receptors may reduce M1 microglial polarization and neuroinflammation by influencing ER-mitochondrial interactions and function in models of stress-induced hypertensive rats and valproic acid-induced autistic rats (Ooi et al., 2021; Mohamed et al., 2025). Consistently, exogenous activation of sigma receptors significantly reduces microglial activation following traumatic brain injury (TBI) (Shi et al., 2022). Surprisingly, the sigma-1 receptor inhibition significantly reduced cortical microglial proliferation in a mouse model of chronic osteoarthritis pain, thereby alleviating depression-like behaviors (Carcole et al., 2019). This demonstrates that the role of sigma receptors in microglia may vary depending on the context of depression. In addition to microglia, the effects of sigma receptors on astrocytes have been observed. In mouse models with depression-like symptoms, blocking the sigma-1 receptor in astrocytes triggers depression-related behaviors through NF-κB-driven neuroinflammation, which is alleviated by sigma-1 receptor agonists (Wang et al., 2024). Coincidentally, activating the astrocytic sigma-1 receptor significantly alleviates lipopolysaccharide (LPS)-induced depression-like behaviors (Guo L. et al., 2021). These findings indicate the potential of sigma receptors as therapeutic targets for neuroinflammation in depression.

### 4.5 Synaptogenesis/neuronal plasticity: implications for antidepressant efficacy

Depression is a complex mental disorder associated with changes in synaptic plasticity and synaptogenesis, particularly in the prefrontal cortex and hippocampus. Reduction of synapses in brain regions related to mood and cognition is linked to depression, and fast-acting antidepressants such as ketamine could promote synapse formation and repair stress-related synaptic damage (Duman and Aghajanian, 2012). Additionally, changes in spinogenesis in the basolateral amygdala and medial prefrontal cortex contribute to the onset and resolution of depressive episodes as well as the sustained effects of antidepressants such as ketamine and imipramine (Moda-Sava et al., 2019; Leem et al., 2020). Consistently, the expression of BDNF, a protein crucial for synaptic plasticity, is lower in the hippocampus and prefrontal cortex of depressed mice, and antidepressants, such as ketamine, can increase BDNF levels and counteract stress (Duman et al., 2021). These results indicate that targeting synaptic plasticity may be effective in treating depression.

Interestingly, recent studies have highlighted sigma-1 receptor agonists as promising treatments for depression by boosting neuroplasticity. Activation of the sigma-1 receptor improved anxiety and depression-like behaviors within a week by reversing estrogen withdrawal-induced reductions in hippocampal dendritic complexity and spine density (Ren et al., 2024). Consistently, co-administration of selective sigma-1 and 5-HT1A receptor agonists enhances neurogenesis and synaptic plasticity in the dorsal hippocampal dentate gyrus, potentially producing an antidepressant effect in chronically stressed mice (Ren et al., 2025). Additionally, several studies have demonstrated the role of sigma receptors in LTP, a crucial representation of synaptic plasticity. A study showed that sigma receptor activation might counteract the impaired LTP induced by corticosterone, olfactory bulb resection, or CaMKIV-deficiency and have antidepressant benefits, in which the Akt/GSK-3β/β-catenin pathway enhanced neurogenesis and increased phosphorylation of CaMKII and GluA1 might be involved (Moriguchi et al., 2015; Kaminska et al., 2000; Moriguchi et al., 2013). Consistently, male mice lacking the sigma-1 receptor show depression-like symptoms (Zhang B. et al., 2017; Voronin et al., 2020; Salaciak and Pytka, 2022), reduced field excitatory postsynaptic potential (fEPSP) slope, impairments in LTP and LTD, and decreased NMDAR NR2B phosphorylation. Administration of an NMDA agonist improved LTD-related behavior and depressive symptoms. Indicating that sigma-1 receptor deficiency affects nNOS activity and NO production via NMDA receptor dysfunction, reducing GABAAR-mediated inhibition and leading to depression-like phenotypes (Zhang B. et al., 2017; Qin et al., 2022). Taken together, sigma receptor modulators hold considerable promise for enhancing depression treatment by facilitating synaptogenesis and neuronal plasticity (Yang et al., 2019; Hashimoto, 2013; Klawonn et al., 2017; Ishima et al., 2014).

## 5 Ligand classes and clinical progress

### 5.1 Clinically approved antidepressants with sigma receptor activity

#### 5.1.1 Tricyclic antidepressants

TCAs are pharmacological agents that inhibit norepinephrine and serotonin reuptake, thereby increasing the concentration of these neurotransmitters at synaptic junctions. Previous studies have shown that TCAs interact with sigma receptors (Paschos et al., 2009). Opipramol, a compound approved in Europe for the treatment of depression and anxiety, has a high affinity for sigma-1 receptors and a low affinity for sigma-2 receptors. Subchronic administration of opipramol reduced sigma-2 receptor expression, whereas prolonged administration of opipramol resulted in a reduction in sigma-1 receptor mRNA in the nucleus accumbens (Holoubek and Muller, 2003). Additionally, the antidepressant effects of another antidepressant, igmesine, depend on sigma receptors, as evidenced by its ability to reduce immobility time in the forced swim test (FST) and chronic forced swim (CFS) tests, effects that are inhibited by Sigma-1 receptor antagonists and sigma-1 knockout mice (Villard et al., 2011). Desipramine also exerts modulatory effects on sigma receptors by affecting their expression in glial cells (Barg et al., 1991).

#### 5.1.2 Selective serotonin reuptake inhibitors

SSRIs are a class of antidepressants that inhibit serotonin reuptake, aligning with the monoamine hypothesis that depression is linked to reduced monoamine levels (Skuza et al., 2011). Fluvoxamine, a notable SSRI that shows significant binding to sigma receptors (Hindmarch and Hashimoto, 2010), alleviates methamphetamine-induced anxiety behaviors through sigma-1 receptor activation (Zhang et al., 2023). In rats with chronic mild unpredictable stress (CUMS)-induced MDD, fluvoxamine exhibits antiarrhythmic effects via sigma-1 receptor-dependent mechanisms (Guo Y. et al., 2021). Other SSRIs that interact with sigma receptors include, sertraline which inhibits LTP in rat hippocampal slices through inverse agonism at the sigma-1 receptor (Izumi et al., 2024); fluoxetine which shows high affinity for sigma receptors and enhances nerve growth factor (NGF)-induced neurite outgrowth (Ishima et al., 2014); and citalopram which modulates the response of embryonic thalamic axons to Netrin-1 through sigma-1 receptor activation (Bonnin et al., 2012). Notably, paroxetine does not exhibit sigma-1 receptor agonistic effects (Sugimoto et al., 2012). It is interesting to identify the SSRIs that functions through sigma receptors and analyze the common structural features of these SSRIs, which may help developing novel SSRIs with higher selectivity or efficacy.

#### 5.1.3 Monoamine oxidase inhibitors

MAOIs are recognized for their effectiveness in treating depression by increasing brain monoamines through the inhibition of their degradation (Yang et al., 2019). Compounds such as selegiline and rasagiline display neuroprotective properties by stabilizing mitochondrial function, inhibiting apoptotic pathways, and promoting neurotrophic factor expression. These effects are partly mediated by sigma receptors (Behl et al., 2021; Naoi and Maruyama, 2010). Clorgyline shows a high affinity for the sigma binding site in the C57BL/6 J mouse brain (Itzhak and Kassim, 1990), whereas deprenyl reduces sigma-1 receptor-mediated behavioral sensitization to cocaine (Tsai et al., 2015). Elucidating the roles of sigma receptors in MAOIs may help in developing novel MAOIs with minimal adverse effects, such as the “cheese effect” while enhancing neuroprotective outcomes (Finberg, 2014; Schapira, 2011).

#### 5.1.4 Neurosteroid and acetylcholinesterase inhibitor

Neurosteroids have emerged as promising antidepressant agents, especially for the treatment of postpartum depression (McEvoy et al., 2018; Rupprecht et al., 2023; Kargbo, 2023). Crystallographic studies have revealed that progesterone and dehydroepiandrosterone sulfate (DHEAS) interact with the sigma-1 receptor through hydrophobic interactions, which might contribute to DHEAS-enhanced presynaptic glutamate release in the prefrontal cortex (Fu et al., 2024; Chen et al., 2017). Another neurosteroid, dehydroepiandrosterone (DHEA), increases GLT-1 activity and promotes the migration of hippocampal astrocytes through sigma-1 receptor-mediated PKC activation, thereby providing protection against excitotoxicity (Dong et al., 2007). Donepezil, a central acetylcholinesterase inhibitor that acts as a sigma-1 receptor agonist, has also been reported to alleviate depression in patients with Alzheimer’s disease, probably due to its direct binding to sigma-1 receptors in the human brain, as evidenced by PET scans (Carotenuto et al., 2022; Devanand et al., 2018).

### 5.2 Novel sigma receptor ligands in preclinical development

#### 5.2.1 Sigma-1 receptor ligands

YL-0919 is chemically known as 1-(1-benzyl-4-hydroxypiperidin-4-ylmethyl)-2(1H)-pyridinone hydrochloride. Intragastric administration of YL-0919 alleviates depressive symptoms, as assessed by the FST and Tail Suspension Test (TST), without increasing suicide risk or causing weight gain (Sun et al., 2019). Consistently, YL-0919 could enhance the firing of glutamatergic pyramidal neurons in the mPFC (Yan et al., 2024), highlighting the necessity of considering cell type and/or brain region when studying the mechanisms underlying YL-0919 therapy. Additionally, in the mPFC, YL-0919 promotes neuronal remodeling and restores mitochondrial function (Li J. F. et al., 2023) and reduces NF-κB-induced neuroinflammation in depressed mice with sigma-1 receptor knockdown (Wang et al., 2024). In the hippocampus, YL-0919 boosts cyclic adenosine monophosphate (cAMP) signaling (Ran Y. H. et al., 2018) and activated mTOR pathway, which contributes to its effects of decreasing swimming retention time and feeding latency (Ran Y. et al., 2018). However, to the best of our knowledge, whether sigma receptors in different cell types or brain regions function differently during YL-0919 therapy is largely unknown.

Interestingly, sigma receptors in astrocytes may be potential targets for YL-0919 therapy. For example, inhibiting sigma-1 receptors on ventral hippocampal astrocytes led to anxiety and depression-like behaviors and blocked YL-0919’s antidepressant effects in stressed mice. Consistently, knockdown of sigma-1 receptors hinders the ability of YL-0919 to improve mitochondrial function and BDNF expression in astrocytes (Li et al., 2025). As for microglia, previous studies showed that the administration of YL-0919 partially reversed PLX3397-induced microglial depletion in the mPFC of mice and alleviated depressive symptoms (Chang et al., 2023); however, whether these effects were attributed to sigma receptors on microglia needs further exploration.

SA-4503, chemically known as 1-(3,4-dimethoxyphenethyl)-4-(3-phenylpropyl) piperazine dihydrochloride, acts as a sigma-1 receptor agonist (Matsuno et al., 1996; Kobayashi et al., 1996; Senda et al., 1996). SA-4503 exhibits antidepressant effects through various mechanisms, including (1) formation of sigma-1 receptor-5-HT1A complexes, showing neuroprotective effects that were inhibited by sigma-1 receptor antagonist (Ren et al., 2025; Skuza and Rogoz, 2007); (2) synergistic interactions with NMDA receptor antagonists (Wang et al., 2007; Skuza and Rogoz, 2006), including enhancement of the antidepressant effects of ketamine, which were partially counteracted by BD1407 (Ma et al., 2024); (3) improvement of hippocampal LTP (Moriguchi et al., 2015); and (4) enhancement of cardiac function, showing its efficacy in treating depressive-behavior associated with heart failure (Liu X. et al., 2019; Liu et al., 2018; Shinoda et al., 2016). Current research has predominantly focused on the adjunctive use of SA-4503 with antidepressants, whereas studies on its independent neurobiological mechanisms remain limited, thus offering avenues for future research.

SKF-83959, chemically known as 6-Chloro-7,8-dihydroxy-3-methyl-1-(3-methylphenyl)-2,3,4,5-tetrahydro-1H-3-benzazepine, is a triple reuptake inhibitor with antidepressant properties (Liu et al., 2009; Jiang et al., 2014; Fang et al., 2013). As a potent allosteric modulator of the sigma-1 receptor, SKF-83959 inhibits the calcineurin/GSK3β pathway in the hippocampus, thereby alleviating depression-like symptoms in mice with epilepsy (Guo et al., 2022). SOMCL-668, a novel sigma-1 receptor modulator derived from SKF-83959, also showed rapid antidepressant effects in the CUMS model. However, in contrast to the inhibition of SKF-83959 on hippocampal GSK3β signaling, SOMCL-668 is reported to simultaneously increase GSK3β phosphorylation in the hippocampus (Guo et al., 2022), indicating the distinct mechanisms underlying SOMCL-668 and SKF-83959 therapy. However, their effects were inhibited by sigma-1 receptor antagonists. SCH-23390, an analog of SKF-83959, serves as a positive allosteric modulator of the sigma-1 receptor (Zhang G. F. et al., 2024), although its antidepressant effects require further validation. Understanding the mechanisms of action of SKF-83959, SCH-23390, and SOMCL-668, along with their structural frameworks, may inform future antidepressant drug development.

Other sigma-1 receptor agonists, such as SKF-10047, PRE-084, and igmesine (Zhang K. et al., 2017; Wang et al., 2019; Skuza and Rogoz, 2009; Ito et al., 2012; Urani et al., 2001), have shown antidepressant effects in behavioral paradigms, although additional laboratory data are needed. Sigma-1 receptor antagonists, including PD-144418, BD-1047, and BD-1063, have been found to mitigate “mania-like” behaviors without causing depression-like behaviors (Sanchez-Blazquez et al., 2018), and the sigma-1 receptor antagonist E-52862 has demonstrated antidepressant effects in osteoarthritis mouse models, which may be mediated by the inhibition of cortical microgliosis (Carcole et al., 2019). In pharmaceutical research, newly synthesized pyrimidine thioethers (5c, 5e, and 5f) have shown antidepressant and cognitive enhancement properties, potentially through interactions with sigma receptors (Fioravanti et al., 2023). Understanding the structure–activity relationships and employing computer-aided design can aid in the discovery of novel sigma receptor-binding agents and the mechanisms underlying their antidepressant effects.

#### 5.2.2 Sigma-2 receptor ligands

The sigma-2 receptor complex has been implicated in autophagy, cholesterol synthesis, progesterone signaling, and receptor stabilization, all of which are associated with depression (Izzo et al., 2020). Consistently, mice deficient in the sigma-2 receptor/Tmem97 exhibit reduced depression-like behavior, particularly in females (Hong et al., 2024). However, research on sigma-2 receptors and their ligands in the context of depression is relatively limited compared to studies on sigma-1 receptors. Lu 28–179, a selective sigma-2 receptor ligand, demonstrated antidepressant effects at a dosage of 1.0 mg/kg administered subcutaneously per day in a rat model of chronic mild stress-induced depression (Sanchez and Papp, 2000). Ibogaine, a compound derived from African psychedelic flora, shows a high affinity for sigma-2 receptors (Ki: 90.4 and 250 nM) but a lower affinity for sigma-1 receptors (Ki: 9310 nM) (Mach et al., 1995; Bowen et al., 1995). A single intraperitoneal administration of ibogaine (20–40 mg/kg) has been shown to produce antidepressant-like effects in rats, which are attributed to increased BDNF mRNA levels in the prefrontal cortex (Rodri Guez et al., 2020). Based on the pharmacophore of ibogaine, several novel small-molecule compounds have been discovered and shown to be effective in alleviating depression-like symptoms in murine models (Singh et al., 2023; Cameron et al., 2021). Importantly, a case report suggested the effectiveness of ibogaine in reducing depressive symptoms in bipolar depression (Fernandes-Nascimento et al., 2022). These results suggest that the sigma-2 receptor is a promising therapeutic target for depression and merits further investigation for drug development.

Despite the identification of promising ligands, the sigma-2 receptor remains underexplored, a paradox largely due to several interconnected limitations. First, the sigma-2 receptor had not been cloned for an extended period and was believed to be an 18–22 kDa protein enriched in lipid rafts (Crawford et al., 2002). It was not until 2017 that the sigma-2 receptor was identified as TMEM97, a protein involved in cholesterol regulation (Alon et al., 2017). This delayed validation of the sigma-2 receptor molecular identity significantly hindered early progress. Consequently, initial studies on sigma-2 receptor ligands primarily relied on *in vitro* cell lines (Barg et al., 1994), as researchers lacked genetic tools (e.g., knockout models) to investigate the roles of sigma-2 receptors and their ligands in depression, which were assessed through behavioral testing. Furthermore, the expression of the sigma-2 receptor varies across heterogeneous cell lines (Barg et al., 1994; John et al., 1998; John et al., 1999), and this inconsistency has also impeded consensus on the therapeutic potential of the sigma-2 receptor in depression, even for well-characterized ligands. Third, the sigma-2 receptor is part of the progesterone receptor membrane component (PRMC) family, which shares structural homology with other cholesterol-sensing proteins (Chu et al., 2015; Hiranita, 2016). Consequently, sigma-2 receptor ligands may cross-react with other PRMC family members, such as PGRMC1, or with cholesterol transporters, potentially resulting in off-target effects that obscure sigma-2 receptor-specific signaling (Zeng et al., 2019; Riad et al., 2020). Until recently, no sigma-2 receptor ligands with absolute selectivity were available, thereby limiting researchers’ ability to ascertain whether the observed antidepressant effects are attributable to sigma-2 receptor activity rather than off-target binding.

Additionally, sigma receptor ligands can also bind to multiple targets. As previously discussed, numerous sigma receptor ligands, particularly those developed in the early stages, including fenfluramine, opipramol, and donepezil, often exhibit limited selectivity (Rao et al., 1990; Martin et al., 2020; Kato et al., 1999). For example, (1) Donepezil is known to interact with acetylcholinesterase and α-synuclein, which enhances LTP (Mandai et al., 2020); (2) Opipramol has the potential to engage with dopamine receptor, thereby enhancing dopamine metabolism in the striatum and other brain regions (Rao et al., 1990); (3) Ketamine could directly bind to NMDA receptor to activate the BDNF-mTOR pathway (Kim et al., 2024); and (4) Sertraline could directly bind to SERT, which could in turn enhances synaptic plasticity (Ferres-Coy et al., 2016). The off-target effects of these ligands make it difficult to precisely determine the role(s) of the sigma receptors in these biological processes. Additional experiments base on advanced methodologies such as network pharmacology, computational modeling and omics technologies may help us understand whether the observed effect of sigma receptor ligand attributes to its action on sigma receptors, other targets or both.

### 5.3 Sigma ligands in clinical trials

Currently, sigma ligands have attracted considerable interest in clinical trials in treating depression, including:

SA-4503, a sigma-1 receptor agonist, has shown antidepressant effects in animal studies. In 2007, a Phase II clinical trial assessed the safety and efficacy of SA-4503 in individuals with major depression, involving 150 participants over an eight-week period. However, the results of this trial have not yet been published (ClinicalTrials.gov Identifier: NCT00551109). Opipramol, which binds to both sigma-1 and sigma-2 receptors (Volz and Stoll, 2004), has been found to alleviate hot flashes and depressive symptoms in postmenopausal women compared to placebo (van Lith and Motke, 1983). In a double-blind study with 17 participants aged 28–60 years, the combination of opipramol and baclofen significantly reduced polysubstance use disorder and depressive symptoms while also increasing DHEA-S levels and the DHEA-S to cortisol ratio, indicating potential therapeutic benefits for depression in patients with substance use disorder (Bareli et al., 2021). Fluvoxamine, a selective serotonin reuptake inhibitor, exhibits agonistic effects on sigma-1 receptors. A two-month study involving patients with MDD found that fluvoxamine alleviated depressive symptoms and lowered interleukin-6 (IL-6) levels, with these effects partially mediated by sigma receptor activity (ClinicalTrials.gov Identifier: NCT04160377) (Li X. et al., 2023). A study on fluvoxamine maleate for the treatment of depression in children remains unpublished [ClinicalTrials.gov Identifier: NCT00353028]. AXS-05, a combination of dextromethorphan and bupropion, also acts as a sigma-1 receptor agonist. In a Phase III trial with 327 patients with MDD, a 6-week treatment with AXS-05 proved superior to placebo in the Montgomery-Asberg Depression Rating Scale (MADRS) scores, with 39.5% of participants achieving remission (ClinicalTrials.gov Identifier: NCT04019704) (Iosifescu et al., 2022). Another trial also showed that AXS-05 had greater efficacy than bupropion in reducing MADRS scores over 6 weeks (ClinicalTrials.gov Identifier: NCT03595579) (Tabuteau et al., 2022). A trial comparing AXS-05 with a placebo for depression recurrence is forthcoming (ClinicalTrials.gov Identifier: NCT04608396). Indeed, the efficacy of AXS-05 in treating depression has been reviewed in a previous systematic review of five studies, demonstrating that AXS-05 significantly reduced depression severity within 2 weeks, with effects lasting for 12 months, suggesting that it is a rapid-acting option for MDD (Akbar et al., 2023). Dextromethorphan, a component of AXS-05, exhibits rapid antidepressant effects via sigma receptors in animal models (Nguyen et al., 2014). A phase IIa clinical trial with 20 patients with treatment-resistant depression assessed the combination of dextromethorphan and quinidine. Over a 10-week period, the Montgomery-Åsberg Depression Rating Scale (MADRS) scores decreased by 13.0 points, and the Quick Inventory of Depressive Symptomatology-Self Report (QIDS-SR) scores decreased by 5.9 points, indicating preliminary efficacy (Murrough et al., 2017). An ongoing study is currently recruiting participants to investigate depression-related neuropsychiatric changes in patients with HIV who are starting treatment with dolutegravir in combination with TDF/FTC (ClinicalTrials.gov: NCT06787976). YL-0919 is in phase 2 trials, showing antidepressant effects and multitarget properties in preclinical studies (ClinicalTrials.gov: NCT03404466, NCT03739632, and NCT04598607) (Table 2).

**Table 2:** Sigma receptor ligands in clinical trials.

Compounds	Clinical phase	Patients No.	Condition	Outcome	ClinicalTrials.gov Identifier
SA4503	Phase II	150	MDD	Unreleased	NCT00551109
Opipramol	Pilot study	17	Polysubstance use disorder (PSUD)	Significant improvement in depressive symptoms	Not registered
Fluvoxamine	Phase II	150	Depression	Relieved	NCT04160377
	Phase IV	90	MDD	Unreleased	NCT00353028
AXS-05	Phase III	327	MDD	Partial remission	NCT04019704
	Phase II	97	MDD	Decrease in MADRS	NCT03595579
	Phase II	44	MDD	Unreleased	NCT03595579
Dextromethorphan	Phase IIa	20	Treatment-resistant depression	Decrease in MADRS and QIDS-SR	Not registered
	Phase IV	140	Patients with HIV	Unreleased	NCT06787976
YL-0919	Phase I/II	42	MDD	Unreleased	NCT03404466
	Phase II	240	MDD	Unreleased	NCT03739632
	Phase I	36	MDD	Unreleased	NCT04598607

## 6 Conclusion and prospect

Sigma receptors play crucial roles in the development of depression. Recent studies on sigma receptor ligands have made significant progress, with sigma-1 receptor agonists demonstrating antidepressant effects. Many therapeutic benefits of antidepressants are linked to sigma receptors, and drugs targeting these receptors are currently undergoing clinical trials. Sigma receptor ligands affect monoaminergic neurotransmission and synaptic plasticity through intracellular translocation. Differences in binding affinities may explain the varied antidepressant effects, highlighting the need for personalized treatment strategies. The complex nature of sigma receptors offers the potential for depression treatment, and the development of high-affinity ligands could effectively address depression and its comorbidities. Although research on sigma receptor ligands is promising, there are still significant gaps. The lack of large-scale clinical trials limits the confirmation of efficacy and safety in patients with depression, as most evidence comes from preclinical studies. Research must clarify the long-term effects on the body and the mechanisms of action, especially regarding neurotransmitter interactions. Although research on sigma-2 receptors is limited, previous studies may guide the development of pharmacological agents. The development of specific sigma receptor ligands is essential, as existing ligands exhibit off-target effects. Future research should focus on developing high-specificity ligands using computational methods and high-throughput screenings.
